# Am I getting through? Surveying students on what messages they recall from the first day of STEM classes

**DOI:** 10.1186/s40594-021-00306-y

**Published:** 2021-08-06

**Authors:** Clara L. Meaders, Lillian G. Senn, Brian A. Couch, A. Kelly Lane, Marilyne Stains, MacKenzie R. Stetzer, Erin Vinson, Michelle K. Smith

**Affiliations:** 1 Section of Cell and Developmental Biology, Division of Biological Sciences, University of California, San Diego, La Jolla, CA 92093 USA; 2 Department of Ecology and Evolutionary Biology, Cornell University, E145 Corson Hall, Ithaca, NY 14853 USA; 3 School of Biological Sciences, University of Nebraska, Lincoln, NE 68588 USA; 4 Department of Biology Teaching and Learning, University of Minnesota, Minneapolis, MN 55455 USA; 5 Department of Chemistry, University of Virginia, Charlottesville, VA 22904 USA; 6 Department of Physics and Astronomy, University of Maine, Orono, ME 04469 USA; 7 Maine Center for Research in STEM Education, University of Maine, Orono, ME 04469 USA

**Keywords:** Undergraduate, First day, STEM courses, Non-content Instructor Talk, Classroom observations, Messaging

## Abstract

**Background:**

The first day of class helps students learn about what to expect from their instructors and courses. Messaging used by instructors, which varies in content and approach on the first day, shapes classroom social dynamics and can affect subsequent learning in a course. Prior work established the non-content Instructor Talk Framework to describe the language that instructors use to create learning environments, but little is known about the extent to which students detect those messages. In this study, we paired first day classroom observation data with results from student surveys to measure how readily students in introductory STEM courses detect non-content Instructor Talk.

**Results:**

To learn more about the instructor and student first day experiences, we studied 11 introductory STEM courses at two different institutions. The classroom observation data were used to characterize course structure and use of non-content Instructor Talk. The data revealed that all instructors spent time discussing their instructional practices, building instructor/student relationships, and sharing strategies for success with their students. After class, we surveyed students about the messages their instructors shared during the first day of class and determined that the majority of students from within each course detected messaging that occurred at a higher frequency. For lower frequency messaging, we identified nuances in what students detected that may help instructors as they plan their first day of class.

**Conclusions:**

For instructors who dedicate the first day of class to establishing positive learning environments, these findings provide support that students are detecting the messages. Additionally, this study highlights the importance of instructors prioritizing the messages they deem most important and giving them adequate attention to more effectively reach students. Setting a positive classroom environment on the first day may lead to long-term impacts on student motivation and course retention. These outcomes are relevant for all students, but in particular for students in introductory STEM courses which are often critical prerequisites for being in a major.

**Supplementary Information:**

The online version contains supplementary material available at 10.1186/s40594-021-00306-y.

## Introduction

The first day of class is commonly used by instructors to set the tone for the semester. For example, studies of the first day of class in psychology courses have revealed that non-content activities, such as reciprocal interviews where an instructor interviews students about their goals and expectations for the course and then uses these responses to set expectations for the students, and having a positive first day of class can impact student perceptions of their instructors, course satisfaction, and motivation (Hermann & Foster, 2008; Hermann et al., 2010; Wilson & Wilson, 2007). As such, there are many recommendations about how to approach the first day (e.g., creating connections and motivating students; Anderson et al., 2011) and varying ways that science, technology, engineering, and mathematics (STEM) instructors structure and emphasize messaging (Lane et al., 2021). However, despite the availability of recommendations about effective practices during the first day of class, there is a noted lack of empirical data about the relevance of this day for students (Mancini, 2017). In particular, little is known about whether students detect messages conveyed during the first day of class in their STEM courses. Here, we define detect as the ability for students to reflect on what occurred in class and indicate whether an instructor conveyed a particular message.

Prior studies have focused on what instructors do and say on the first day of class, using combinations of student surveys, instructor interviews, and observations. In one study, students in a communications course answered a survey where they were asked to think about the first day of class in another course and identify the instructional practices used by the instructor (Friedrich et al., 1993). Collectively, the students returned surveys detailing the instructional practices from 145 courses. The students reported general instructional trends (e.g., that instructors typically introduce course policies and procedures and most students have positive experiences); however, because of the study design, student responses were not associated with particular instructors. In a second study, instructor interviews were used to explore the instructional practices of 18 instructors identified by academic vice presidents as excellent teachers (Iannarelli et al., 2010). Inductive thematic analysis of the interviews revealed that these instructors all generally had four objectives during the first day of class: (1) communicating course expectations, (2) learning about students, (3) introducing the instructor, and (4) establishing the tone or atmosphere of the course. These two studies established general trends but were unable to connect how much time instructors spend on specific first day topics with messages students detect. To better understand how instructor actions impact students, we designed a study that links what messages instructors send on the first day of class and whether they are detected by students.

### Conceptual framework

This study was guided by a conceptual framework that synthesizes ideas from the non-content Instructor Talk Framework (Harrison et al., 2019; Seidel et al., 2015) and Vygotsky’s sociocultural theory (SCT; Vygotsky, 1978). Specifically, we used the non-content Instructor Talk framework to classify the types of messages instructors make on the first day. This framework focuses on language that is not directly course-specific content but is instead used to establish learning environments (Harrison et al., 2019; Seidel et al., 2015). For example, instructors can share personal stories or explain why they use specific teaching practices (e.g., small group discussion), which are advocated approaches to creating positive learning environments and reducing student anxiety (Hsu & Goldsmith, 2021). The non-content Instructor Talk framework has also been adapted to different contexts including the characterization of non-content talk instructors use to support students during stressful events such as the pandemic (Seah et al., 2021).

Previous work suggests that non-content Instructor Talk occurs more frequently during the first day of class compared to subsequent class periods (Seidel et al., 2015). Furthermore, the non-content Instructor Talk framework was adapted by Lane et al., 2021 to document the non-content information shared by 23 instructors from across STEM disciplines on the first day of class. Observations of the first day of class showed that although instructors varied in their approaches, instructors could be classified into two clusters: one cluster that dedicated higher amounts of time to STEM content on the first day and a second that spent little time on STEM content and covered more policies and basic course information.

To frame how non-content Instructor Talk used on the first day of class may impact subsequent student learning and engagement within a course, we used the lens of sociocultural theory (SCT) (Vygotsky, 1978). One of the core tenets of SCT is that social interactions form the basis of learning (Vygotsky, 1978) and that teachers guide classroom discourse to promote student learning (Scott, 1998). Instructor discourse, norms, and instructional practices provide a model that informs students’ integration into the classroom community as well as into the broader scientific discipline (Forman & McCormick, 1995). Starting on the first day of class, instructors set the social context for scaffolded learning through non-content talk and activities that help students get to know each other (Iannarelli et al., 2010). Social contexts and classroom climates influence student perceptions of their courses at large, their instructors, their peers, and their own experiences, and may mediate longer-term student attitudes, motivation, and achievement within a course (Adelman & Taylor, 2002; Barr, 2016; Evans et al., 2009). Indeed, in an undergraduate biology course, positive classroom climates set by instructors were associated with higher student course persistence and course satisfaction (Barthelemy et al., 2015).

### Current study rationale and research questions

While classroom climates can be created in many ways throughout a term, non-content messaging during the first day of class represents a common way that instructors communicate their support and teaching beliefs to students at the outset of the course and may be a key tool to promote positive social contexts for student learning. However, the specific impacts of non-content talk on the first day of an undergraduate STEM class remain unexplored. Non-content messaging may be communicated in different ways by instructors, and a key first question is whether students detect these non-content messages. There is a precedent for measuring what students detect as previous studies have established that student perceptions can provide reliable and valid indications of instructional practices (Ehman, 1970). Similarly, students and instructors have shown high agreement with one another regarding the occurrence of many scientific teaching practices in the classroom (Durham et al., 2018).

Identifying the extent to which students detect messages given by their instructors provides a variety of benefits for instructors. For example, given that instructors may cover a number of topics, knowing how much time is necessary for students to detect a message could help instructors prioritize what and how long to cover specific messages. If there are types of messaging that are important to instructors but which students are not detecting as important, this can help instructors better plan what to emphasize in class. Finally, student recollections of non-content messages from the first day of class provide a window into what students take away from the first day besides basic information and content coverage.

In this study, we observed the first day of class for 11 STEM instructors at two universities, and surveyed students within the courses. We asked (1) what are the non-content messages shared by faculty on the first day of class and (2) how likely were students to detect the non-content messaging? Because the non-content Instructor Talk framework used for the observations provided the basis for student survey questions, this study provides a novel link of instructor practices during the first day of class and what students detect.

## Methods

### Participants

We recruited faculty participants through professional development programs at two research intensive, doctoral granting universities. Collectively, these faculty taught 11 introductory and upper-level in-person courses across seven STEM disciplines as defined by the National Science Foundation (biology, chemistry, forestry, economics, mathematics, statistics, physics) during the spring 2020 semester, before universities in the USA were closed because of the COVID-19 pandemic. Four of the 11 instructors had been observed in previous years for a separate study focused on instructional practices used during the first day of class (Lane et al., 2021).

### Class period structure and Instructor Talk analysis

We recorded the first day of class for all 11 courses by either placing a single video camera at the back of the lecture hall or using an audio recorder at the front of the lecture hall. We then generated class period transcripts using the Otter transcription and editing platform (Otter.ai).

We used two strategies for coding the first day of class (outlined in Table 1). We first coded for class period structure. Previous work has found that differences in class period structure are primarily driven by how much time instructors spend either on STEM content or policies and basic information (Lane et al., 2021). We coded every second of a class period for the three mutually exclusive categories: STEM content, course logistics (i.e., instructional strategies, instructional technologies, and policies and basic information), and all other first day topics (e.g., goals and relevance of the course) (Additional file 1: Appendix S1). We were able to code class period structure at intervals of a second because it was straightforward to identify start/stop time points in the transcripts.

**Table 1:** Summary of data sources and coding strategies

What was analyzed	Purpose	Data source	Coding details
Class period structure	Categorizing first day structure	Researcher observations of classroom audio/video transcripts	Coded observations for (1) STEM content, (2) course logistics, and (3) all other first day topics at 1-s intervals*
Non-content Instructor Talk	Document the use of non-content Instructor Talk	Researcher observations of classroom audio/video transcripts	Coded observations for presence/absence of nine categories of non-content Instructor Talk at 1 min intervals*
	Identify student detection of non-content Instructor Talk	Student responses to survey questions	Analyzed student survey responses about the presence/absence of features associated with non-content Instructor Talk categories within a class period

*Codebooks are available in Additional file 1: Appendix S1 and S2.

Authors CLM and LGS reviewed two (18%) of the class period transcripts separately. Their percent agreement on the categorization of each second for each of the three videos was > 97%, and author CLM coded the remaining nine videos. We calculated the total number of seconds instructors spent on any of these three categories and divided each number by the total number of seconds in the class. We then used the heatmap.2 function in the gplots package (Warnes et al., 2020) in RStudio (RStudio Team, 2016) for hierarchical cluster analysis, which generated two clusters of instructors: one cluster who focused more time on STEM content coverage, and another cluster who spent lower amounts of time on STEM content coverage.

In addition to coding for class period structure, we coded the same transcripts for positively phrased non-content Instructor Talk (Table 1). Positively phrased non-content Instructor Talk is language that is framed to promote classroom environments, goals, or student learning (Harrison et al., 2019; Seidel et al., 2015). This framework has been previously used to establish a coding process for documenting Instructor Talk on the first day of class (Lane et al., 2021). Because the precise start/stop points for non-content Instructor Talk are more difficult to identify when compared to class period structure, we used 1 min intervals similar to a segmented observation protocol such as COPUS (Smith et al., 2013), and each minute was coded for the presence/absence of each code (Additional file 1: Appendix S2). The coders, CLM and LGS, separately reviewed the transcripts and entered codes into a spreadsheet. The coders then met and discussed each minute, re-watched intervals as necessary, and came to consensus for how to code the intervals. Each code was organized into nine overarching categories of messaging (Additional file 1: Appendix S2). Three of the nine categories are based directly on the overarching categories *sharing personal experiences, promoting diversity in STEM,* and *being explicit about the nature of STEM* from Lane et al., 2021; Seidel et al., 2015. The six remaining categories also include original codes from the non-content Instructor Talk framework, but these codes have been reorganized from their original overarching categories into new groups. This change allowed us to assess the presence or absence of additional non-content messaging that previously had been grouped with other categories (Additional file 1: Appendix S2). To visualize patterns of non-content Instructor Talk, we constructed boxplots using the ggplot2 package (Wickham, 2016). We then used the t.test function in R to identify if there was a difference in the means of the amount of time instructors dedicated to non-content Instructor Talk between the two groups of instructors: instructors who structured the first day of class with higher STEM content coverage and lower STEM content coverage.

### Documenting the prevalence of non-content Instructor Talk

In all 11 STEM courses, we coded for a potential of nine categories of non-content Instructor Talk. The amount of time dedicated for a category within each course was rounded to the nearest percentage and binned as covered in at least 4% of 1 min intervals, covered less (greater than 0% but less than 4% of 1 min intervals), or not covered (0% of observed 1 min intervals). We chose 4% of 1 min intervals as a cutoff, because 4% of a 50 min class period is the equivalent of two minutes of class time, and we posit that two minutes is the minimum amount of time we can use to measure in-depth coverage of a category. Although we coded at 1 min intervals, within a single 1 min interval an instructor could state a single sentence that would be coded but was not a substantial message. Using a 4% cutoff and the accompanying two minutes of class time from 50 min class periods requires that at a minimum there were two intervals in which a category was coded. The two minutes could occur consecutively (meaning the instructor likely spent the majority of the minutes on that category) or could be dispersed throughout the class period (meaning the instructor may have said two short phrases).

### Development of the student survey and validity evidence

To determine whether students were detecting non-content Instructor Talk messages on the first day of class, we surveyed them during the first week of the semester (Table 1). We took steps during the student survey development process to optimize survey validity, and we explored relationships with other variables to gain further validity evidence (AERA, 2014).

#### Steps taken to increase validity during survey development

For the student survey, we based questions on the non-content Instructor Talk framework (Harrison et al., 2019; Seidel et al., 2015) and observation codebook (Additional file 1: Appendix S2), which had been developed from a prior study focused on capturing Instructor Talk on the first day of class using video observations (Lane et al., 2021). This approach helped support content validity by ensuring that the survey covered the range of messages that might be sent by an instructor on the first day. Two authors, CLM and MKS, adapted codes into forced-choice, check-all-that-apply questions, with an option of “none of the above” (Additional file 1: Appendix S3). Each of the checklist options mirrored language used in the non-content Instructor Talk framework codes used by the researchers and we took care to minimize educational jargon. Although there are negatively phrased Instructor Talk categories developed by Harrison et al., 2019, we chose to survey students only on positively phrased Instructor Talk so as to not inadvertently negatively impact student attitudes through our survey questions (Wilding et al., 2016). To increase face validity, the remaining authors, who have experience using the non-content Instructor Talk framework to conduct classroom observations, provided feedback on the survey and it was iteratively revised.

#### Evidence based on relations to other variables

Another important approach to establishing validity examines the relationships between the instrument, in our case the student survey, to other external variables to provide construct validity. There are several ways to evaluate construct validity, but we chose to correlate student survey responses with actual observation data to determine the extent to which student perceptions reflected classroom events. To mitigate potential observer bias, we completed all the classroom observations and analyses before exploring the student survey data.

The comparisons between the course observation and student survey data are described in the Results sections entitled *Students’ ability to broadly detect non-content messaging* and *Students’ ability to detect non-content Instructor Talk messaging in specific courses*. Here, we explored the following questions: (1) When observers report a non-content Instructor Talk case, do students indicate that they detected the relevant message on the student survey? (2) When observers do not report a non-content Instructor Talk case, do students report that they did not detect the relevant message? Affirmative answers to both questions provide validity evidence for the student survey based on other variables.

### Distribution of the student survey

Instructors introduced the survey and distributed the survey link using lecture slides, email, course management system messages, and verbal announcements. Each time they introduced the survey, the instructors clarified that the study was being conducted by researchers independent from the course and that they would not see individual student responses. Students completed the surveys online, outside of class. The surveys were open for one week, starting after the end of the first class period. One course disseminated the survey to their students one week after the first class period. Instructors varied in providing incentives for students to complete the survey, with the majority of instructors offering participation points.

Total course enrollment was 2130 students (Additional file 1: Appendix S4). We received an initial 1571 responses, and then removed student responses that were (1) not complete, (2) duplicate responses from students who completed the survey two or more times for a single course, and (3) from students who indicated that they had not attended the first day of class. This process left a total of 1429 responses and a 67% final overall response rate, with a range of 39–88% across courses, including 905 responses from University 1 and 514 responses from University 2 (Additional file 1: Appendix S4).

### Comparison of student and researcher observations

For every student response, we calculated a binary “match” variable: whether they matched or did not match with observers regardless of how long an instructor spent on a non-content Instructor Talk category. To be categorized as a match, students and observers had to each report a category of non-content Instructor Talk as present or absent in a course. If a student reported a category as present that the coders observed as absent, or reported a category as absent that the coders observed as present, the student was identified as a “non-match.” We calculated the Pearson correlation coefficient to identify if there was a correlation between students’ matching with observers and the amount of time instructors dedicated to non-content Instructor Talk.

Additionally, students self-reported on demographic variables (gender, underrepresented racial/ethnic minority (URM) student status, and first-generation college student status) (Additional file 1: Appendix S3), and we collected information from the survey software about how soon after class students completed the survey (within 1 h of class on the first day, before their second class period, or after their second class period). Completion of the demographic questions was optional, and 1340 of the 1429 students completed all of the demographic questions. Overall demographic information is shown in Additional file 1: Appendix S4. We analyzed student responses from students who identified as male or female students, students who were URM students or non-URM students, and students who were first-generation college students or continuing generation college students. We input the demographic variables and survey completion time as independent variables, the match variable as a dependent variable, and students as a random effect in a logistic regression using the lme4 (Bates et al., 2015) and MuMin (Bartoń, 2020) packages in R.

For each course, we calculated the percent of students who reported that a category was present or absent during the first day of class, and binned what students detected according to standards set by Landis & Koch, 1977. If greater than 80% of students reported that a category was present, this indicated that the majority of students within a course reached consensus that the category was present. If between 60 and 80% of students reported that a category was present, this indicated moderate consensus by students: while the majority of students detected a message, it was to a lesser extent. Similarly, students could reach higher levels of consensus (greater than 80%) or moderate consensus (between 60 and 80% of students) that a category was absent. Finally, if between 40 and 60% of students reported that a category was covered, this indicated that the students within a course did not reach consensus about a category, as ~ 50% of students had reported a category “present” and 50% “absent.” We compared the case metrics for the amount of time instructors dedicated to categories (using the 0%, 0–4%, and ≥ 4% bin) with the student survey consensus levels.

## Results

### Instructors vary in the amount of STEM content and non-content talk on the first day

**Fig. 1:**
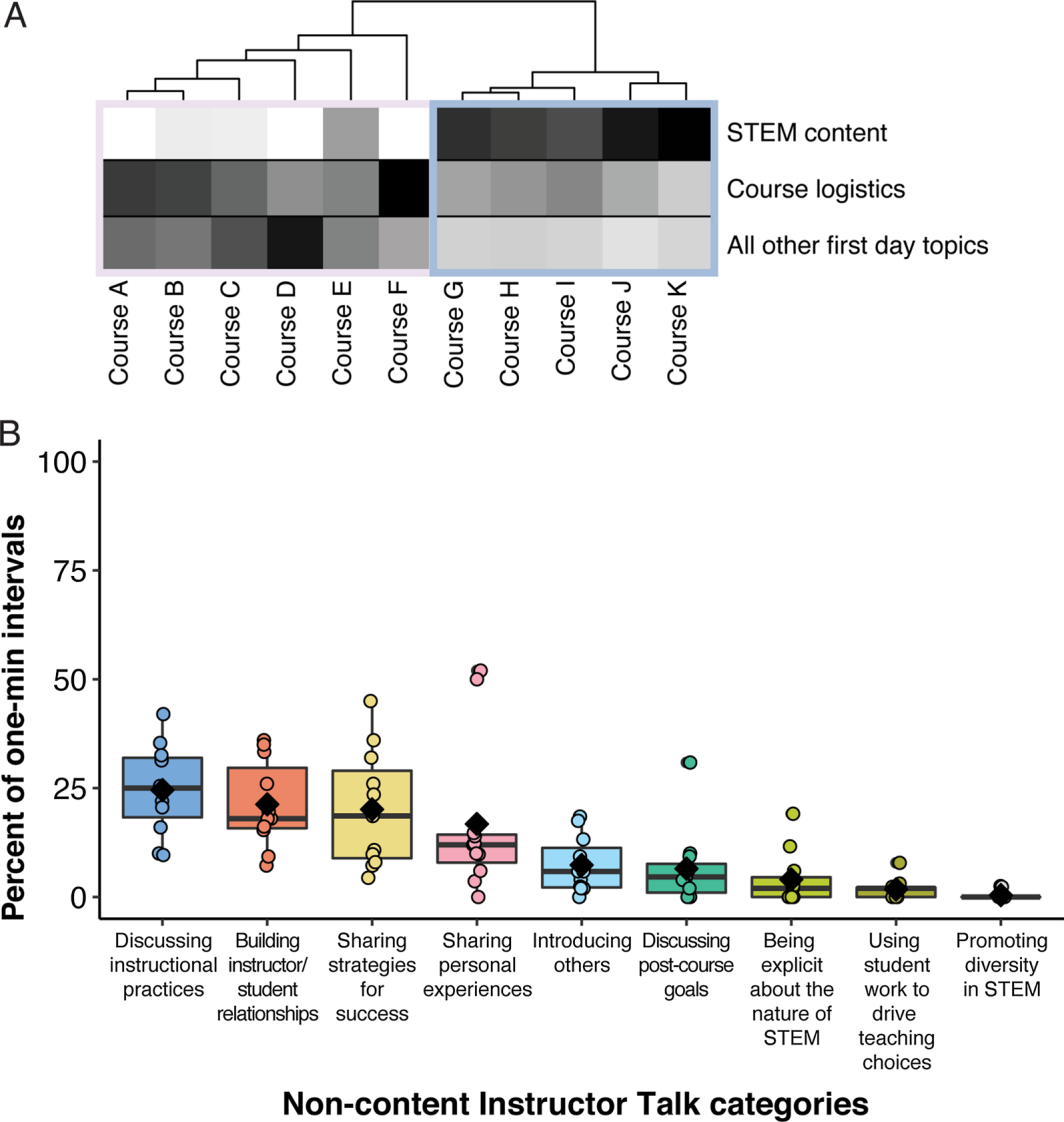
Structure and non-content talk on the first day of class. **A** Heatmap of the amount of time instructors dedicated to course logistics, STEM content, or all other first day topics. Instructors are ordered based on dendrogram clusters, with the lower STEM content coverage cluster outlined in light purple and the higher STEM content coverage cluster outlined in light blue. **B** Boxplot of the percent of time instructors spend on each non-content Instructor Talk category. Each box represents the interquartile range (IQR). Whiskers represent 1.5 times the IQR. Lines within each box represent the median, and diamonds represent the mean for that category. Circles represent the data points from the 11 instructors and are included to show the spread of time within each category

Observations of instructors across 11 STEM courses revealed similarities and differences in approaches to the first day of class. We calculated the total amount of time instructors dedicated to STEM content, course logistics, and all other first day topics, and used hierarchical cluster analysis to generate two clusters of instructors. Similar to Lane et al., 2021, we found that instructors varied in how they structured the first day of class (Fig. 1A). The primary difference between the two groups of instructors is that one cluster of six instructors spent lower amounts of time on STEM content coverage (lower STEM content coverage) and another cluster of five instructors focused more time on STEM content coverage (higher STEM content coverage, Fig. 1A). The two clusters differed significantly (t-tests with Bonferroni corrections) along all three aspects of class period structure, with lower STEM content coverage instructors spending more time on course logistics and all other first day topics (Additional file 1: Appendix S5).

We then explored overall patterns of non-content Instructor Talk for the 11 instructors (Fig. 1B; Additional file 1: Appendix S6). The most common category of non-content Instructor Talk was *discussing instructional practices,* with instructors spending an average of 25 ± 10% of 1 min intervals on this category*.* On average, all other categories were present in less than 25% of 1 min intervals. We identified a trend that instructors who spent lower amounts of time on STEM content had higher percentages of 1 min intervals dedicated to *building instructor/student relationships* and *sharing strategies for success* (Additional file 1: Appendix S5)*.* This trend was also observed in a previous study that focused on the first day of class (Lane et al., 2021). However, after applying a Bonferroni correction for multiple hypothesis testing for the t-tests, we did not identify significant differences in the percent of 1 min intervals dedicated to various categories of non-content Instructor Talk between instructors who spent lower and higher amounts of time to STEM content (Additional file 1: Appendix S5).

### Students’ ability to broadly detect non-content messaging

Because instructors vary in their approaches to the first day of class, a natural next question is whether students detect the messages shared by their instructors. For each student survey response, we compared their detection of messaging to observer data using a “match” variable, which was determined by identifying whether each student matched with observers regarding the presence or absence of each non-content Instructor Talk category in their courses. We found that as the mean implementation of categories of non-content Instructor Talk increased, the percent match between observers and student survey results also increased (Additional file 1: Appendix S5). The Pearson's correlation coefficient between these values is significant (*r* = 0.89 *p* = 0.001). In other words, the more a category was addressed, the more students agreed regarding whether it occurred.

To determine if students with different demographics detected non-content Instructor talk messages differently, we used a logistic regression to identify if student demographic variables such as gender, URM-status, or first-generation student status impacted student detection of non-content Instructor Talk using the match variable as a dependent variable. We also explored whether the time students completed the survey (within 1 h of class on the first day, before their second class period, or after their second class period) influenced the match variable. The best-fitting model did not include gender, first-generation student status, or the time when the students completed the survey, indicating that these factors did not explain variation in the match variable. However, the odds of aligning with observers were 1.2 times greater for URM students than for non-URM students, (*p* = 0.01) (Additional file 1: Appendix S5). We visualized the raw data using a cross-tabulation and found that 85% of the URM students matched observers versus 83% of the non-URM students (Additional file 1: Appendix S5). We disaggregated the data by non-content Instructor Talk category and found that the difference in matching is primarily driven by two categories: *using student work to drive teaching choices* and *being explicit about the nature of STEM.* For these two categories, URM students aligned with observers at higher rates than non-URM students (Additional file 1: Appendix S5).

### Students’ ability to detect non-content Instructor Talk messaging in specific courses

**Fig. 2:**
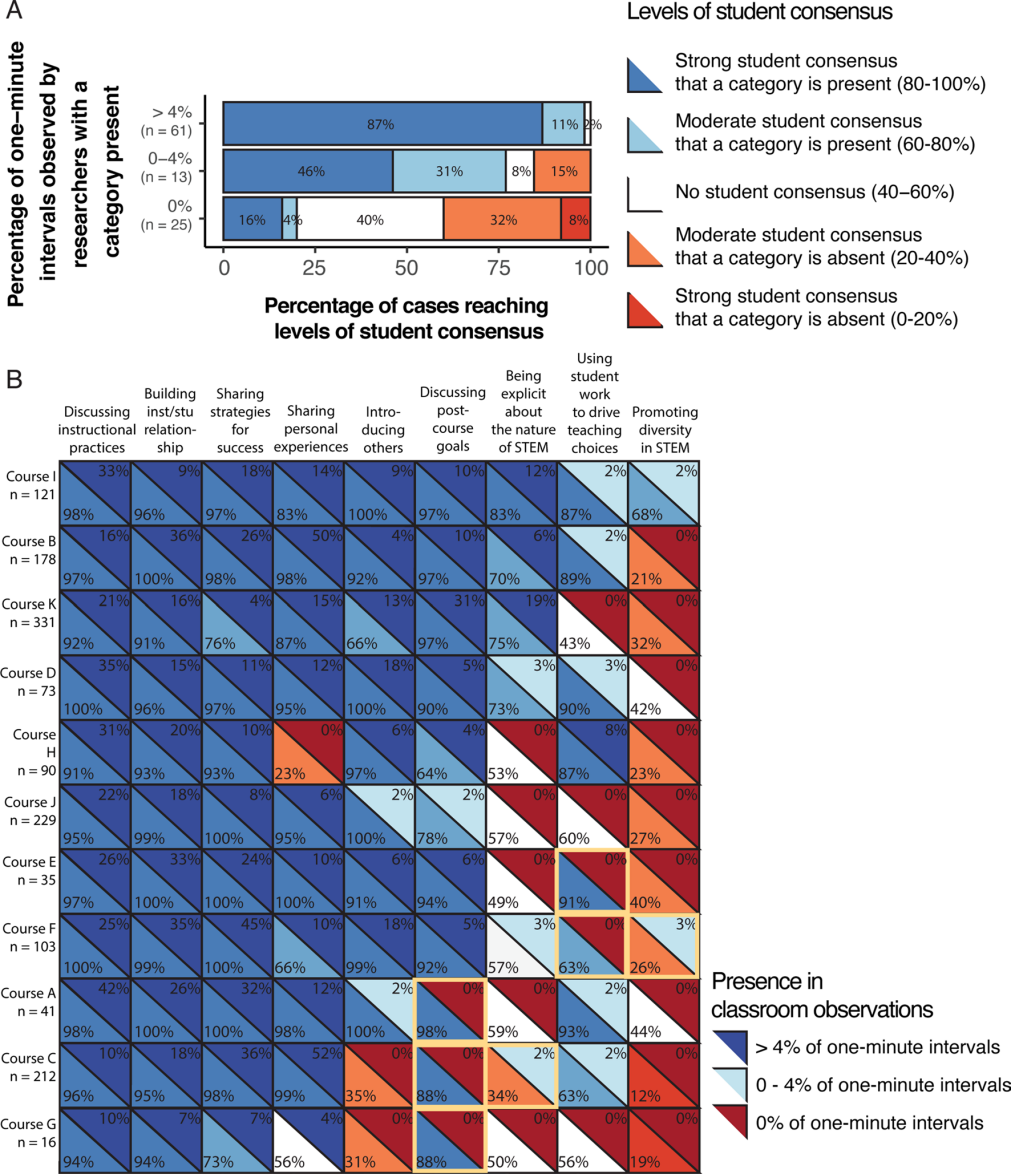
Comparison of student consensus within each course that individual categories were covered during the first day of class and the percentage of 1 min intervals observed by researchers. **A** Each stacked bar represents the total percentage of categories from across all 11 courses where researchers observed at least 4%, 0–4%, or 0% of 1 min intervals dedicated to a category. Within each stacked bar, the colors represent the percentage of cases where students from a course reached varying levels of consensus that a category was present or absent. **B** Detailed summary of the cases depicted in (**A**). The upper right triangles depict the percentages of 1 min intervals observed by researchers for each course, shaded according to the bins shown in the key to the right. The lower left triangles depict levels of student consensus that a category was present or absent, shaded according to the levels shown in the key above. Courses are ordered from top to bottom according to the number of categories with strong student consensus that a category was present, and the number of researcher observations of high frequency (at least 4% of 1 min intervals). The number of student responses from each course is included below each course number. Yellow borders indicate noteworthy cases of observer–student disagreement

In order to identify if student detection was consistent across courses we compared our observations with levels of student consensus regarding the presence or absence of categories from within each of the 11 courses. With nine categories and 11 courses, this resulted in 99 cases for us to compare student consensus with the established percent of 1 min intervals dedicated to a category from our observations. For this analysis, we binned each case of a category occurring within a course based on the frequency of researcher observations (in ≥ 4%, between 0 and 4%, or in 0% of 1 min intervals). We found that in 98% of cases where instructors dedicated at least 4% of 1 min intervals to a category, students within those courses reached strong or moderate consensus that the categories were present (Fig. 2A). In cases where instructors covered a category but at lower frequencies (between 0 and 4%), 77% of the time students reached strong or moderate consensus that the categories were present. Student consensus varied in cases where we did not observe instructors covering a category in a class (0%). In 40% of cases, students accurately noted with strong or moderate consensus that a category was absent. However, students did not reach consensus in 40% of cases and inaccurately reported that the category was present in 20% of cases.

A closer look at how researcher observations compared to levels of student consensus across categories and courses revealed trends in the data. First, three categories that were present at high frequencies consistently had strong levels of student consensus: *discussing instructional practices, building instructor/student relationships,* and *sharing strategies for success*. Students from all 11 courses reached moderate or strong consensus that these categories had been covered (Fig. 2B), which also provides validity evidence for the student survey.

We reviewed course transcripts and observed that instructors used a variety of strategies when covering higher frequency categories. For example, the instructor from Course E covered *building instructor/student relationships* by using messaging focused on empathy at intervals throughout the class period, telling students “I know every faculty [goes over this], but again, I have a resource for you. If anything's going on in your life, I want to be very supportive of you. But also know I do have to report it legally to the university. So that's the disclaimer there. But anything you need, I'm happy to help.” Student survey results showed that 100% of the students noticed that the instructor from Course E addressed the category. The instructor from Course A spent several consecutive minutes providing evidence for why using active learning is important when they were *discussing instructional practices,* telling students “So why should we use active learning? Well, it turns out that students in traditional lectures are 1.5 times more likely to fail. Okay? So active learning helps students, less students fail in active learning. Students in traditional lecture classes have lower grades, one standard deviation less. So not only do you not fail as much if you're using active learning, you get better grades in active learning as well. And so this is again, this was a meta study of 200 studies. And so there's a lot of research out here that there's a positive aspect to doing this active learning. A lot of instructors around the University are trying to use active learning.”

Another trend we identified was that for lower frequency (between 0 and 4%, or the equivalent of one minute of a 50 min class period) cases, the methods instructors used to convey specific categories appeared to influence levels of student consensus. When the instructor referenced a category directly related to student experiences in the course, such as *introducing others* and *using student work to drive teaching choices,* students readily detected the categories even at low frequencies (Fig. 2B). In one example, the instructor from Course J dedicated one minute to having instructional assistants introduce themselves, and 100% of students reported that *introducing others* had occurred (Table 2).

**Table 2:** Example non-content Instructor Talk from instructors who dedicated between 0 and 4% of 1 min intervals to messaging

Level of student consensus	Description
Strong student consensus—category is present	Course J, *Introducing others* “So I wanted to pass the mic, the figurative mic, over to our [instructional assistants] and have them introduce themselves and say a little bit about themselves.” This instructor had instructional assistants introduce themselves and speak to the class for a 1 min interval.
Moderate student consensus—category is present	Course C, *Using student work to drive teaching choices* “So I'm participating in a class. The group is participating in a set of meetings that I go to about once a month with a group of people who are teaching similar classes. They're not only [subject specific scientists], the rest of them are in science, computer science or stats, and we are meeting to talk about data that we utilize in our classrooms to make our teaching more effective. And so the first assignment that you have is to click on that link and fill out a qualtrics survey.” This instructor discussed being a member of a faculty learning community, and introduced the first day of class survey.
No student consensus	Course F, *Being explicit about the nature of STEM* “So if you are interested in [topic 1], the study of [subject] is going to help you. Not only because [topic 2], but also at a large level, if you're interested in what's going on in [topic 1], knowing what’s going on at [topic 2] can help you understand some of those, some of those influences.” This instructor discussed the collaborative nature of one topic and the broader STEM field.

Each row details an example quotation from an instructor who dedicated 0–4% of 1 min intervals to a category, and the level of student consensus reached regarding the presence of that category.

Categories such as *using student work to drive teaching choices* and *being explicit about the nature of STEM* may require either more time or certain language for a majority of students to detect them when they are covered at lower frequencies. For example, students reached a moderate consensus that *using student work to drive teaching choices* was covered in Course C (Fig. 2B). In that course, the instructor introduced that they were participating in a data-driven faculty learning community and introduced the survey, but did not explicitly mention that they would use the findings from the survey to inform their teaching (Table 2). On the other hand, students did not reach consensus when the instructor from Course F dedicated 1 min to mentioning that understanding the subject could help with understanding other subjects (Table 2). The observers coded this statement about collaborations as *being explicit about the nature of STEM* but students provided mixed results on the survey (Fig. 2B). The disconnect may be due to the emphasis on other fields in this statement rather than other scientists as described in the student survey (Additional file 1: Appendix S2).

When cases of non-content Instructor Talk were observed at lower frequencies, students were less likely to detect these messages on the survey (Fig. 2B *e.g.,* *promoting diversity in STEM*). In our study, two instructors dedicated between 0 and 4% of 1 min intervals to *promoting diversity in STEM*, and what students detected differed for each instructor. In the first case, the instructor from Course I stated, “And I just want to mention that we strongly believe in diversity, if you're a diverse group of students in all sorts of ways, including your academic background, a lot of your freshmen, but a fair number of you are also second semester seniors. A lot of you are science majors but a lot of you are health. In fact, we have people from, I think, four or five different colleges. And here, we're estimating like 36 different majors. Really diverse, diverse, in terms of backgrounds. You name it, we value that.” The majority of students from this course reported that their instructor had covered the category *promoting diversity in STEM* (Fig. 2B)*.* In the other case, the instructor from Course F dedicated a 1 min interval to *promoting diversity in STEM,* saying “We really want you to succeed, we want to keep you in our majors. I really believe in science and I believe in scientists, and a diverse outlook of scientists. We want to keep you here.” However, the majority of students from this course did not report that this category had been covered (Fig. 2B).

Although infrequent, we identified 12 cases where students did not reach consensus. Ten of these cases occurred when a category was absent (Fig. 2B). Notably, six out of 12 cases where students did not reach consensus occurred in the category *being explicit about the nature of STEM*, indicating that messaging in this category may be presented in ways not fully captured by the student survey.

Finally, we identified seven cases where students reached consensus about the presence or absence of a category, but where their consensus did not match what was observed in class (Fig. 2B; highlighted with yellow borders). There were five cases where the majority of students within a course had reported that their instructor had spent time *discussing post-course goals* or *using student work to drive teaching choices* while the observers had not coded for the presence of these two categories (Fig. 2B). We reviewed transcripts of the courses with these discrepancies and identified language that students may have used to inform their responses. The *discussing post-course goals* category includes language focused on how a course can help prepare students for life beyond college, and how a course relates to a student’s career. In the three cases where students marked this category present, instructors had indirectly discussed post-course goals without using language specific enough for the definition (Table 3). Similarly, in the two cases where students reported *using student work to drive teaching choices* as present, the researchers observed the instructors introduce a pre-test or survey, but did not observe the instructors discussing how the feedback would inform their teaching (Table 3).

**Table 3:** A description of the seven cases where students reached agreement, but their agreement did not match what was observed in class

Issue	Description
Category absent but students marked as present	Course C, *Discussing post-course goals* "This is a class of [subject] and one class is going to give you a taste, but you're not going to learn very much, you're not going to learn as much as you need to do if you're actually going to go out there and do [subject]. Which case reading the book is gonna get you closer to that, and then taking intermediate [subject], will get you closer, and then taking a master's level [subject] will be even closer. And in finally taking a PhD class in [subject] might make you capable after five to 10 years of additional research of doing [subject] policy." The instructor discussed how little students would learn in the class. **We did not code this as post-course goals, as the quotation was remarking on how *little* students would gain from the course, not how much it would fit in with future curricula**
	Course A, *Discussing post-course goals* "half of you guys, that's the main reason to take this course is because you have to take [course]." The instructor mentioned most students were taking the course as a requirement. **We did not code this as post-course goals, as while it acknowledged how the course fit in with curricula the instructor did not talk about any specifics of how the course fit with student goals**
	Course G, *Discussing post-course goals* “While I'm thinking of it [a former student] was on campus yesterday…she mentioned that one of the students who's in the class had asked about if there are any work opportunities this coming summer and said yes, I plan on having two internships… and to encourage more students to contact her. She plans on getting the information out on those internships to me soon. So you can wait until the announcement comes through and if you're interested in this type of work, I encourage you to apply for those internships.” **We did not code this as post-course goals as while the instructor advertised a post-course opportunity, the language used in messaging did not include how the course prepared students for the work**
	Course E, *Using student work to drive teaching choices* “you'll be doing a pretest and a post test” **We did not code this as using student work to drive teaching choices, because the instructor did not describe how the test would be used**
	Course F, *Using student work to drive teaching choices* “There are two participation surveys and five points each one is first day questions and one is for working with our [TA] program. We're always interested in your guys' insights.” **We did not code this as using student feedback to inform teaching choices, because the instructor did not describe how the survey feedback would be used**
Category present but students marked as absent	Course F, *Promoting diversity in STEM* “We really want you to succeed, we want to keep you in our majors. I really believe in science and I believe in scientists, and a diverse outlook of scientists. We want to keep you here.” **We coded this as promoting diversity in STEM, but we also coded it as the instructor demonstrating desire for students to learn or succeed**
	Course C, *Being explicit about the nature of STEM* “So all of us are making decisions all the time and [subject] looks at how we make those decisions, and what the outcome of those decisions result. So it's the study of decisions under conditions of scarcity” **This was coded, but was the last phrase stated in the last minute of class as students were beginning to pack their bags**

The remaining two cases occurred where the research team observed that categories were present, but where the majority of students reported the categories had not been covered. In one case, students did not report observing their instructor *being explicit about the nature of STEM,* but researchers had in fact observed the instructor discussing this category during the last minute of the first day as students were packing their bags and may not have heard the category covered (Table 3). The other case involved an instructor *promoting diversity in STEM* using non-specific language. Overall, the seven examples of disagreement and 12 examples where students did not reach consensus collectively represented a fraction (19%) of the cases explored in this study, and in the majority of cases students and observers were aligned.

## Discussion

In this study, we aimed to (1) characterize how instructors approached non-content messages on the first day and (2) identify the extent to which students detected non-content Instructor Talk used by their instructors during the first day. This work builds on prior work identifying non-content Talk across biology courses (Harrison et al., 2019; Seidel et al., 2015) and characterizing non-content Talk on the first day of class from STEM courses (Lane et al., 2021). Our study is one of the first to directly link quantitative observations of non-content Instructor Talk with student recollections. This link opens the possibilities of larger scale studies focused on instructional practices using student reports through surveys, such as the one described here and future surveys that probe other meaningful components of non-content Instructor Talk. We discuss our findings and the implications they have for educators and future research questions.

Recommendations for the first day of class typically include some form of communicating course expectations, learning about students, introducing the instructor, and establishing the course tone (Anderson et al., 2011; Iannarelli et al., 2010). The 11 instructors in our study dedicated time to practices that fell into these categories, but differed in the amounts of time dedicated to each as well as the way the messaging was framed (Fig. 1). We found that faculty tended to approach the first day in one of two ways: either lower STEM content coverage but higher coverage of course logistics (e.g., policies and basic information) and classroom norms and culture (e.g., introducing the instructor), or higher STEM content coverage and an accompanying lower coverage of course logistics and classroom norms and culture (Fig. 1A). These two clusters align with previous research on the first day of class across STEM courses (Lane et al., 2021). Previous observations of four of the 11 instructors contributed to the cluster analysis from Lane et al., 2021. In conjunction with new observations of the four instructors, the addition of seven other instructors supports that our cluster results apply to a broader group of instructors.

In general, students accurately detected highly covered non-content messaging. Across all categories, when instructors dedicated at least 4% of 1 min intervals from the first day of class to a category, the majority of students in a class detected the messages (Fig. 2A). In our study, instructors dedicated the most time to *discussing instructional practices, building instructor/student relationships,* and *sharing strategies for success* (Fig. 1B). Students reached moderate and strong consensus that these categories had been covered by their instructors (Fig. 2B). Instructors may invest their time in high-frequency categories in different ways, either through sporadic statements throughout a class period or with longer chunks of a class period dedicated to one category. Regardless, it appears that as long as instructors discuss a category of importance across 4% of in-course time (or roughly two minutes in a 50 min class period) during the first day, students will likely detect the message.

Other research has found that students accurately report estimates of how often teaching strategies were used, but that when teaching strategies were present at lower frequencies what students detected was more mixed (Durham et al., 2018). Our data suggests that there may be certain categories of non-content Instructor Talk that students readily detect at low levels (0–4% of 1 min intervals), and other categories that require either extended amounts of time or specificity with language. For example, some categories, such as *introducing others* and *using student work to drive teaching choices,* were detected by students even when they were present in only one minute of class time. This result may be due to instructor choices regarding the messaging. For example, in Course J the instructor devoted 2% of 1 min intervals for instructional assistants to introduce themselves to students (Table 2). While this is a low amount of time, the shift in speaker and hearing directly from instructional assistants may explain why 100% of students from this course reported that *introducing others* had occurred. Similarly, students may pay more attention when instructors discuss pre-tests or class surveys (Fig. 2B). In our study, students often readily detected *using student work to drive teaching choices* when it was present at lower frequencies. The two cases where students had reported this category as present while the observers had recorded it as absent involved courses where instructors introduced pre-tests and/or surveys, but did not discuss how the results would inform their teaching (Table 3). Overall, the presence or absence of introductions or assessments may be more readily noticed by students.

Interestingly, we identified 10 cases where a category was absent but students were mixed in their reporting, with approximately half of students in those courses reporting that the categories had been covered, and an additional five cases where the majority of students reported that an absent category had been covered (Fig. 2B). These cases occurred when instructors spent time *discussing post-course goals, being explicit about the nature of STEM, using student work to drive teaching choices,* and *promoting diversity in STEM.* Our transcripts reveal that students may be more generously interpreting what counts as these messages (Table 3), but other explanations could include that some students may be assuming that a particular message may have occurred, or trying to help their instructors appear more favorable even though we stressed that the study was being conducted by researchers independent from the course. Conducting interviews with students to learn more about the moments they are referring to in their responses could clarify these discrepancies.

### Why does student detection of non-content Instructor Talk on the first day of class matter?

We used sociocultural theory (SCT) when framing the broader goals of this study (Vygotsky, 1978) and posit that student learning is impacted by non-content Instructor Talk starting on the first day of class. Our results support that instructors attend to the sociocultural communities within their courses to different degrees through the use of non-content talk and that overall students detect the messages shared by their instructors. Students in introductory courses hold a variety of concerns as well as course anxiety at the beginning of the term (England et al., 2019; Meaders et al., 2020), and the first day of class and language used by instructors provides an opportunity to directly address student questions and alleviate concerns (Hsu & Goldsmith, 2021; Meaders et al., 2021). Because student perceptions of instructor support are negatively correlated with student anxiety (Schussler et al., 2021) and difficulty in accessing help for course work is a large factor in STEM attrition (Seymour et al., 2019), non-content talk on the first day of class may be a key tool instructors can use to signal support and availability for their students.

Our goal in this study was to establish what students detect regarding messaging they receive on the first day of class. However, whether the first day of class has lasting impacts on STEM student psycho-social metrics and learning remains an open question. Previous work has shown that students form strong first impressions of their instructors within the first 30 minutes of class (Babad et al., 1999). While student impressions are malleable as they collect further information, instructor teaching may impact students’ decisions to drop a course (Babad et al., 2008). In addition, there has been some research exploring the longer-term importance of reciprocal interview activities conducted on the first day of class in psychology courses and about positive first day of class experiences (Hermann et al., 2010; Wilson & Wilson, 2007). Further research is necessary to explore the impacts of first day non-content Instructor Talk on student attitudes toward the course, learning, motivation, and retention within a course or major. The outcomes of these studies are relevant for all students, but in particular for students in introductory STEM courses which are often critical courses for continuing in STEM majors.

### Messaging about promoting diversity in STEM

In this study only two instructors dedicated time to *promoting diversity in STEM* during the first day of class (Fig. 2B). One instructor (Course I) focused on diversity of academic backgrounds, and a majority of students reported that the category had been covered. The other instructor (Course F) stated that they believe in a “diverse outlook of scientists” but did not use other specific language for groups within STEM, and a majority of students reported that the category had not been covered. Indeed, the language an instructor uses may make the difference between students detecting or not detecting a lower frequency message in this category.

With our sample size of 11 courses, it is important to be cautious in making generalizations about how often instructors promote diversity in STEM on the first day of a course. We also recognize that the data in this study were collected in the spring 2020 semester prior to the beginning of the COVID-19 pandemic and that the educational landscape has changed in undergraduate courses. These changes include efforts to promote anti-racism in college classrooms (Ahadi & Guerrero, 2020). To identify if instructors have become more explicit in their messaging about diversity in STEM and whether the messages are detected by students, additional studies are needed.

Going forward, instructors who wish to convey messaging of diversity and inclusion may benefit from identifying explicit language that will be detected by students and by exploring other ways of conveying these messages. For example, Scientist Spotlights (Schinske et al., 2016) and Project Biodiversify (https://projectbiodiversify.org/) provide resources for instructors to highlight the research and academic journeys of diverse scientists. Instructors who include assignments using these or similar resources in their syllabi and discuss the importance of their reasoning for including these assignments in their courses during the first day of class may signal a commitment to promoting diverse scientists. In addition, collecting information about student goals and experiences via surveys or information sheets can serve as an inclusive teaching practice (Killpack & Melón, 2020). Other non-verbal cues such as providing opportunities for students to share information about themselves such as their pronouns may also signal commitments to inclusion in the classroom (Cooper et al., 2020). As STEM instructors develop curricular materials that convey messages detected by students, it will be helpful if they share their resources in open educational resource repositories (e.g., *CourseSource *https://www.coursesource.org/*,* QUBES https://qubeshub.org/, or SERC https://serc.carleton.edu/index.html).

### Limitations

There are a few limitations to consider when interpreting the results from our observation and survey data. First, this study was conducted at two research-intensive institutions and focused on spring semester courses from introductory courses. Researchers who developed the non-content Instructor Talk framework observed that in five out of eight community college biology courses they studied, non-content talk was most prevalent during the first day of class (Harrison et al., 2019). Since the decisions of course structure and types of non-content messaging are relevant in all undergraduate classrooms, we anticipate that instructor approaches to the first day of class will be similar across disciplines at community colleges and other institutions, but it will be important to expand this work to other institutional contexts. Should the student survey be used in more institutional settings as a stand-alone metric without comparisons to observational data, it will be important to interview students about the survey questions to ensure that the statements continue to align to the non-content Instructor Talk framework and are appropriate for a variety of institutional contexts. Interviews will also be helpful to investigate cases where students did not reach consensus such as *being explicit about the nature of STEM* to determine how students think about this category and if additional student survey questions are needed.

An additional limitation is that the faculty in this study were participating in a professional development program. The participants were engaged in a faculty learning community (Cox, 2001) focused on the transition between high school and college, and prior to recording their first day of class, they had engaged in a number of discussions focused on the importance of transparency of instructional practices at the beginning of the semester. In this study, we found that the most common category was *discussing instructional practices* (Fig. 1B). This finding differs from previous work which found that *building instructor/student relationships* was the most common non-content Instructor Talk category (Lane et al., 2021). However, the increased prevalence of *discussing instructional practices* may reflect the impacts of a professional development program. This study could be repeated with different sets of instructors, for example instructors new to teaching or instructional assistants in laboratory or discussion sections.

### Additional future directions

Faculty can use our findings to inform their instructional planning. For example, if faculty aim to convey a certain message to students, discussing that message for at least 4% of 1 min intervals of class time indicates that students will likely detect that message. Faculty who set these types of non-content learning objectives for the first day of class can also use our informal survey to explore student recollection of messaging and identify areas that did not register with students (Additional file 1: Appendix S3).

Further research is needed across a larger sample of instructors at a variety of course levels to determine how instructional practices and messages from the first day of class relate to STEM instructors’ plans for the first day of class, their overall teaching philosophy, and their teaching practices during the remainder of the semester. Future studies should explore alignment between goals for the first day of class, instructor teaching philosophies, and actual practices from the first day of class. This work may reveal differences in approaches to the first day of class based on instructor background, experience, or discipline.

Another future direction is to explore how non-content messages affect students from different demographic backgrounds. We show that most student demographic characteristics do not strongly predict students’ abilities to detect non-content Instructor Talk messages (Additional file 1: Appendix S5). The only significant difference is that URM students matched the observer data slightly better than their non-URM classmates (Additional file 1: Appendix S5). The data suggest that URM students may be more attentive to instructional cues on that first day and possibly more impacted by the non-content Instructor Talk statements an instructor may make. Now that this study has completed the initial step of measuring student detection of non-content Instructor Talk messages, it will be important to examine how messages on the first day of class influence the attitude and performance of students from different demographic backgrounds. Work in K-12 has shown that the greater the number of cultures represented in a classroom of students, the more likely it is that the students will perceive the teacher as a leader and a helper (Levy et al., 1997). Furthermore, students’ perceptions of interpersonal behavior are different based on the interactions between student and instructor demographic backgrounds (Levy et al., 2003). A better understanding of the impact of demographic backgrounds and interactions at the undergraduate level will be informative for designing practices to support all students in the classroom.

In addition, the background of the instructor may also influence student opinions. Notably, several studies have identified student biases in teaching evaluations based on instructor demographics such as gender, age, race, and ethnicity (Bennett, 1982; Chávez & Mitchell, 2020; Joye & Wilson, 2015; MacNell et al., 2015). We are currently exploring how instructor backgrounds influence the impacts of non-content Instructor Talk messaging on the first day of class.

### Conclusion

Our observation- and survey-based study provides the first evidence that students detect higher frequency non-content messaging shared on the first day of class. Additionally, we found that for lower frequency messaging, the category of non-content Instructor Talk messaging and specificity of language used by the instructor may impact what students detect. Instructors can use these findings to help plan how they allocate time during the first day of class and the language they use to address non-content categories of interest.

## Supplementary Information

**Additional file 1:** **Appendix S1.** Codebook for course structure. **Appendix S2. **Codebook for non-content Instructor Talk. **Appendix S3.** Survey questions. **Appendix S4.** Student demographics. **Appendix S5.** Statistical tests. **Appendix S6.** Heatmap of non-content Instructor Talk categories

## Data Availability

The de-identified datasets used and/or analyzed during the current study are available from the corresponding author on reasonable request.

## Abbreviations

STEM: Science, technology, engineering, and mathematics
SCT: Sociocultural theory
