# Supplementary material for: Am I getting through? Surveying students on what messages they recall from the first day of STEM classes
Source: Int J STEM Educ. 2021 Aug 6;8(1):49. doi: 10.1186/s40594-021-00306-y (PMC8344324; doi:10.1186/s40594-021-00306-y)

**Supplemental Materials**

| Supplemental Appendix 1. Codebook for course structure | page 2 |
| --- | --- |
| Supplemental Appendix 2. Codebook for non-content Instructor Talk | pages 3-4 |
| Supplemental Appendix 3. Survey questions | pages 5-7 |
| Supplemental Appendix 4. Student demographics | pages 8-9 |
| Supplemental Appendix 5. Statistical tests | pages 10-12 |
| Supplemental Appendix 6. Heatmap of non-content Instructor Talk categories | page 13 |

**Supplemental Appendix 1. Codebooks for course structure.** We coded the number of seconds instructors dedicated to each of the collapsed codes.

| **Collapsed codes** | **Non-content topics** | **Definition from (Lane et al., 2021)** |
| --- | --- | --- |
| STEM content | STEM content | Course content including students taking content-focused pre-tests. |
| Course Logistics | Instructional strategies | Covering what will occur during a typical class period or week. This non-content topic includes time spent in lab or recitation/breakout sessions. It also includes introducing activities or talking points that will be covered later on the first day. |
|  | Instructional technologies | Ensuring students know how to use classroom technologies (e.g., clickers or course management system). |
|  | Policies & basic information | Covering grading policies, classroom policies, technology policies (e.g., cell phone use), expectations about work related to class (e.g., explaining expected attendance at talks/seminars), safety/emergency policies and procedures, and other similar policies. |
| All other first day topics | Goals & relevance of the course | Explaining why students should care about or choose to take the class and the goals of the class. The instructor may discuss the class in relation to careers, life-long learning, daily life, university requirements, or current events. |
|  | Surveys | Explaining surveys or having students take surveys. This topic is not time spent on pre-quizzes but rather surveys about students’ backgrounds, interests, etc. |
|  | Introduce instructor to the students | Students learning about the instructors or instructors learning about students (e.g., students raising hands to indicate their majors or instructors sharing anecdotes). Teaching/learning assistants were considered instructors for the purpose of this code. |
|  | Introduce students to each other | Giving students opportunities to talk to their peers about things that are not course content. Can also include time the instructor spends reporting to students about the demographics of those taking the course. |
|  | Tips for success | Covering tips and tricks that may help students do well in college overall. This topic is not spent on class-specific policies but strategies  that could apply to any class (e.g., get enough sleep, don’t put off homework until the last minute, etc.). |
|  | Other | Any time spent on the course that did not fit into one of the previous categories. For example, time dedicated to pre-quizzes, or time spent waiting due to technology issues. |

**Supplemental Appendix 2. Codebook for non-content talk categories, including language used in the observer codebook as well as in the student surveys.**

| Non-  content talk category | Observer code definitions | Student survey question | Survey options |
| --- | --- | --- | --- |
| Sharing personal experiences | Information about the instructor’s personal life or their likes and dislikes. This code could include expressing excitement related to having taught this particular class before. | Instructors may introduce themselves on the first day of class. Which, if any, of the following did your instructor talk about on the first day of class? Check all that apply. | Provide information about their lives outside the classroom |
|  | Personal information specifically about the instructor’s college experience and their approach to college and how that echoes students’ experiences. |  | Discuss their experiences when they were undergraduates |
|  |  |  | Share their academic journey |
| Promoting diversity in STEM | Explains why it is important for diverse people to engage in STEM, acknowledges the lack of diversity in STEM, or highlights diverse STEM practitioners. | Did your instructor talk about the importance of having diverse people in science, technology, engineering, and math (STEM) careers? (Check yes or no) | Yes or no |
| Being explicit about the nature of STEM | Explains how STEM is done such as making predictions or that it is iterative, hard, or maybe frustrating. Includes talking about the Nature of STEM. | On the first day of class, instructors may spend time talking about science, technology, engineering, and math (STEM) beyond the scope of the course. Which, if any of the following explanations did your instructor provide about what STEM is like? | That STEM involves making predictions |
|  |  |  | That STEM involves the scientific method |
|  |  |  | That STEM is iterative |
|  |  |  | That STEM requires working collaboratively with other scientists |
|  |  |  | That STEM involves answering unanswered questions |
|  |  |  | That the process of STEM can be challenging and/or frustrating at times |
| Building instructor/  student relationships  **A code was added to this category from the establishing classroom culture category from* Seidel et al., 2015 | Compliments or positive feedback on student work or effort. Statements indicating that all students can succeed given the right effort or tools. | On the first day of class, instructors may spend time talking about their perspective on student success in their course. Which, if any, of the following types of things did your instructor mention on the first day of class? Check all that apply. | The instructor expressed that students can succeed. |
|  | Statements about wanting all students to learn and succeed in this course or college in general. |  | The instructor expressed that they want all students to succeed in their course. |
|  | States that being wrong, providing a wrong answer, or disagreeing with someone is okay or is part of the learning process. Also, may state that instructors can make mistakes. Suggests that the instructor is not interested in hearing the correct answer but instead curious about the students' thought processes. |  | The instructor shared that being wrong, providing a wrong answer, or disagreeing with someone are part of the learning process. |
|  | Considerations of students’ responsibilities and needs both within and outside of class. Descriptions of how the instructor adapts based on students’ needs. Acknowledging challenges that students might face. |  | The instructor acknowledged that students have many responsibilities in other classes and outside of class. |
| Sharing strategies for success **formerly part of the building instructor/student relationships category* (Seidel et al., 2015) | Information about actions or resources that could help students manage their time. | On the first day of class, your instructor may have shared strategies to succeed in the course and/or in college. Which, if any, of the following types of advice did your instructor share? Check all that apply. | Time management strategies |
|  | Information about actions or resources that could help students study more or more effectively. Specifically focuses on test/exam preparation. |  | Study strategies |
|  | Information about actions or resources students can use to seek help including office hours. These are resources related to learning, not student well-being. |  | Discussion of academic resources (e.g. office hours, tutoring, learning strategies center) |
|  | Information about actions or resources related to student well-being. These resources are not about learning but rather about well-being. |  | Discussion of resources for emotional and physical well-being |
|  | Information about actions or resources that seem helpful, but do not fall into one of the other categories. |  | Advice from previous students in the course (e.g. quotations from students or learning assistants sharing tips) |
| Using student work to drive teaching choices  **formerly part of the explaining pedagogical choices category* (Seidel et al., 2015) | Indicates that the instructor uses information from students, either in the form of direct feedback, formative assessments, or pre-tests to make pedagogical choices | On the first day of class, instructors may talk about the importance of student feedback for informing their teaching. Which, if any of the following did your instructors talk about on the first day of class? Check all that apply. | Using a pre-test focused on course content to inform teaching choices |
|  |  |  | Using previous student performance to inform teaching choices |
|  |  |  | Using previous student evaluations to inform teaching choices |
|  |  |  | Using student answers to questions about their backgrounds, interests, and/or concerns to inform their teaching choices |
| Discussing instructional practices  **formerly part of the establishing classroom culture and explaining pedagogical choices categories* (Seidel et al., 2015) | Information on what activities or instructional practices will occur within the classroom throughout the semester. May include how an activity will be done, but not including exam procedures. | On the first day of class, instructors may spend time talking about the instructional practices they use in class, and provide recommendations for how to prepare outside of class. Which, if any, of the following types of course-related things did your instructor mention on the first day of class? Check all that apply. | The instructor explained why they chose to use certain instructional practices in the course (e.g. group work, active learning, clicker-type questions). |
|  | Explains why the instructor chose to structure the course or activities in a way that will help students learn. Includes statements about things required to be done outside of class that will improve activities inside of class. |  |  |
|  | Explains how learning works from a biological, sociological, or psychological perspective. May include references to research on learning. |  | The instructor talked about how people learn. For example saying "we regularly solve problems because it helps you form neurological connections about the content." |
| Discussing post-course goals  **formerly part of the establishing classroom culture and explaining pedagogical choices categories* (Seidel et al., 2015) | Directions to "think like a scientist/engineer/mathematician," such as critical thinking, using data, or being skeptical. Does not need to describe these specifically as STEM skills. | On the first day of class, instructors may spend time explaining how their course will prepare you for your future career path. Which, if any, of the following types of post-course goals did your instructor mention on the first day of class? Check all that apply. | How their course will prepare you to be curious, think critically, and use data. |
|  | Relates course to the real world or a student’s career. Expressions that the class can help prepare students for life beyond college. |  | How their course fits in with the broader curriculum for the major. |
|  |  |  | How their course will provide transferable skills across majors. |
|  |  |  | How their course addresses real world issues. |
|  |  |  | How their course prepares you for future careers |
|  | States that the goal is for students to retain knowledge long-term and not just for the test. Explains how pedagogy helps accomplish this. |  | How their course prepares you to retain the knowledge or subject matter you will gain for the long-term. |
| Introducing other people  **formerly part of the establishing classroom community category* (Seidel et al., 2015) | Positive comments about colleagues’ teaching such as advice, ideas, resources, hard work, caring about students, being good instructors, etc. Also includes giving credit to a group such as a department for good teaching. | Instructors may introduce other people on the first day of class. Which, if any, of the following people did your instructor introduce you to on the first day of class? Check all that apply. | The teaching team. This team could include other co-instructors, teaching assistants, and/or undergraduate learning assistants |
|  | Suggestions that students help each other during in-class activities and for studying outside of class. Also includes breaking down barriers between students by sharing information about themselves with other students. |  | Other students in the course. This could include providing time for you to introduce yourselves to your neighbors |

**Supplemental Appendix 3.** Survey questions

Answer all of the following questions while thinking specifically about [course number].

Instructors may introduce themselves on the first day of class. Which, if any, of the following did your instructor talk about on the first day of class? Check all that apply.

- Provide information about their lives outside of the classroom
- Discuss their experience when they were undergraduates - emphasizing how similar it was from current students' experiences
- Share their academic journey
- None of the above

Instructors may introduce other people on the first day of class. Which, if any, of the following people did your instructor introduce you to on the first day of class? Check all that apply.

- The teaching team. This team could include other co-instructors, teaching assistants, and/or undergraduate learning assistants
- Other students in the course. This could include providing time for you to introduce yourselves to your neighbors
- None of the above

On the first day of class, your instructor may have shared strategies to succeed in the course and/or in college. Which, if any, of the following types of advice did your instructor share? Check all that apply.

- Time management strategies
- Study strategies
- Discussion of academic resources (e.g. office hours, tutoring, learning strategies center)
- Discussion of resources for emotional and physical well-being
- Advice from previous students in the course (e.g. quotations from students or learning assistants sharing tips)
- None of the above

On the first day of class, instructors may spend time talking about their perspective on student success in their course. Which, if any, of the following types of things did your instructor mention on the first day of class? Check all that apply.

- The instructor expressed that students **can** succeed.
- The instructor expressed that they **want** all students to succeed in their course.
- The instructor shared that being wrong, providing a wrong answer, or disagreeing with someone are part of the learning process.
- The instructor acknowledged that students have many responsibilities in other classes and outside of class.
- The instructor gave examples of ways previous students have not succeeded in their course
- None of the above

On the first day of class, instructors may talk about the importance of student feedback for informing their teaching. Which, if any of the following did your instructors talk about on the first day of class? Check all that apply.

- Using a pre-test focused on course content to inform teaching choices
- Using previous student performance to inform teaching choices
- Using previous student evaluations to inform teaching choices
- Using student answers to questions about their backgrounds, interests, and/or concerns to inform their teaching choices
- None of the above

On the first day of class, instructors may spend time talking about the instructional practices they use in class, and provide recommendations for how to prepare outside of class. Which, if any, of the following types of course-related things did your instructor mention on the first day of class? Check all that apply.

- The instructor explained why they chose to use certain instructional practices in the course (e.g. group work, active learning, clicker-type questions).
- The instructor talked about how people learn. For example saying "we regularly solve problems because it helps you form neurological connections about the content."
- The instructor encouraged students to help and seek help from other students.
- None of the above

On the first day of class, instructors may spend time explaining how their course will prepare you for your future career path. Which, if any, of the following types of post-course goals did your instructor mention on the first day of class? Check all that apply.

- How their course will prepare you to be curious, think critically, and use data.
- How their course fits in with the broader curriculum for the major.
- How their course will provide transferable skills across majors.
- How their course addresses real world issues.
- How their course prepares you for future careers.
- How their course prepares you to retain the knowledge or subject matter you will gain for the long-term.
- None of the above

On the first day of class, instructors may spend time talking about science, technology, engineering, and math (STEM) beyond the scope of the course. Which, if any of the following explanations did your instructor provide about what STEM is like?

- That STEM involves making predictions
- That STEM involves the scientific method
- That STEM is iterative
- That STEM requires working collaboratively with other scientists
- That STEM involves answering unanswered questions
- That the process of STEM can be challenging and/or frustrating at times
- None of the above

Did your instructor talk about the importance of having diverse people in science, technology, engineering, and math (STEM) careers?

- Yes
- No

***Optional student demographics questions***

Gender

- Male
- Female
- Not listed above __________
- Prefer not to answer

Race/ethnicity (select all that apply)

- American Indian or Alaska Native
- Asian
- Black or African American
- Hispanic or Latino
- Native Hawaiian or other Pacific islander
- White
- Not listed above __________
- Prefer not to answer

Highest level of education completed by at least one of your parents:

- Did not complete high school
- High school/GED
- Some college (but did not complete college)
- Associate’s degree (2-year degree)
- Bachelor’s degree
- Master’s degree
- Advanced graduate degree (e.g. DVM, MD, PhD)
- Unknown
- Prefer not to answer

**Supplemental Appendix 4.** Student and course demographics.

*Student demographics*

|  | Number of students | % of students |
| --- | --- | --- |
| **University** | | |
| University 1 | 905 | 63% |
| University 2 | 514 | 37% |
| **Current class standing** | | |
| First year | 637 | 44% |
| Sophomore | 448 | 31% |
| Junior | 219 | 15% |
| Senior | 101 | 7% |
| Postbac | 1 | 0.07% |
| Graduate student | 6 | 0.42% |
| Not listed | 6 | 0.42% |
| Prefer not to answer | 2 | 0.14% |
| Left blank | 9 | 0.63% |
| **Gender** | | |
| Male student | 516 | 36% |
| Female student | 877 | 61% |
| Other | 10 | 0.70% |
| Prefer not to answer | 11 | 0.77% |
| Left blank | 15 | 1% |
| **URM*** | | |
| URM student | 267 | 18.7% |
| Non-URM student | 1108 | 77.5% |
| Prefer not to answer | 31 | 2% |
| Left blank | 23 | 1.6% |
| **First-generation student status**** | | |
| First-generation student | 349 | 24.4% |
| Continuing-generation student | 1042 | 73% |
| Unknown | 4 | 0.3% |
| Prefer not to answer | 20 | 1.4% |
| Left blank | 14 | 1% |
| **Students were categorized as URM if they self-reported their race/ethnicity as Black or African American, Hispanic, and/or American Indian and Alaska Native*  ***Students were categorized as first-generation students if neither of their parents had completed a degree from a four-year institution.* | | |

*Course demographics*

| Courses | Course size | N student responses (forced choice check-all-that-apply questions) |
| --- | --- | --- |
| Course A | Medium | 41 |
| Course B | Medium | 178 |
| Course C | Large | 212 |
| Course D | Medium | 73 |
| Course E | Small | 35 |
| Course F | Medium | 103 |
| Course G | Small | 16 |
| Course H | Medium | 90 |
| Course I | Medium | 121 |
| Course J | Large | 229 |
| Course K | Large | 331 |
|  | **2130** | **1429** |
| Student responses per course. Course size: small = 0-49 students, medium = 50-249, large = > 250 | | |

*Student survey completion times*

| Time window when students completed the survey | Number of students | % of students |
| --- | --- | --- |
| Early (within 1 hour after their first day of class) | 74 | 5.1% |
| Middle (later than 1 hour after their first day of class but before their second class period | 863 | 60.4% |
| Late (after the second class period) | 492 | 34.4% |

**Supplemental Appendix 5.** Statistical tests

*T-tests* between the higher STEM content group and lower STEM content group for the percent of time dedicated to STEM content, course logistics, and classroom norms and culture. * represents *p* values less than 0.05, ** represents *p*  values less than 0.01, *** represents *p* values less than 0.001. *P* values have been adjusted for multiple hypothesis testing using a Bonferroni correction.

| Category | *t* value | df | Raw *p*  value | Bonferroni-adjusted *p* value | Mean in Higher STEM content cluster | Mean in Lower STEM content cluster |
| --- | --- | --- | --- | --- | --- | --- |
| STEM content | 9.6 | 9 | 5.04e-06*** | 1.512e-05*** | 61.99 | 6.21 |
| Course logistics | -3.31 | 7.53 | 0.012* | 0.035* | 26.08 | 49.92 |
| All other first day topics | -5.39 | 5.25 | 0.0026** | 0.0078** | 11.93 | 43.87 |

*T-tests* between the higher STEM content group and lower STEM content group for the percent of one-minute intervals dedicated to non-content Instructor Talk topics. ** represents *p*  values less than 0.01. *P* values have been adjusted for multiple hypothesis testing using a Bonferroni correction.

| Category | *t* value | df | Raw *p*  value | Bonferroni-  adjusted *p* value | Mean in Higher STEM content cluster | Mean in Lower STEM content cluster | Number of courses with a category coded |
| --- | --- | --- | --- | --- | --- | --- | --- |
| Discussing instructional practices | 0.38 | 9 | 0.71 | 1 | 23.23 | 25.65 | 11 |
| Building instructor/student relationships | 3 | 8.4 | 0.016* | 0.14 | 14.06 | 27.29 | 11 |
| Sharing strategies for success | 3.6 | 7.28 | 0.008** | 0.07 | 9.6 | 28.88 | 11 |
| Sharing personal experiences | 1.87 | 6.14 | 0.10 | 0.98 | 7.65 | 24.35 | 10 |
| Introducing others | 0.36 | 8.28 | 0.73 | 1 | 6.56 | 7.97 | 10 |
| Discussing post-course goals | -0.85 | 4.61 | 0.44 | 1 | 9.22 | 4.25 | 8 |
| Being explicit about the nature of STEM | -0.95 | 4.43 | 0.39 | 1 | 6.15 | 2.26 | 6 |
| Using student work to drive teaching choices | -0.32 | 4.89 | 0.75 | 1 | 2.03 | 1.51 | 6 |
| Promoting diversity in STEM | -0.08 | 8.58 | 0.94 | 1 | 0.47 | 0.42 | 2 |

Scatterplot visualizing correlations between observer-student agreement and the frequency of non-content messaging. For each category of non-content Instructor Talk, we calculated the mean frequency of implementation across 11 instructors, and the percent of students who matched with observations. The Pearson’s correlation between these values is significant (r = 0.89 p =0.001).


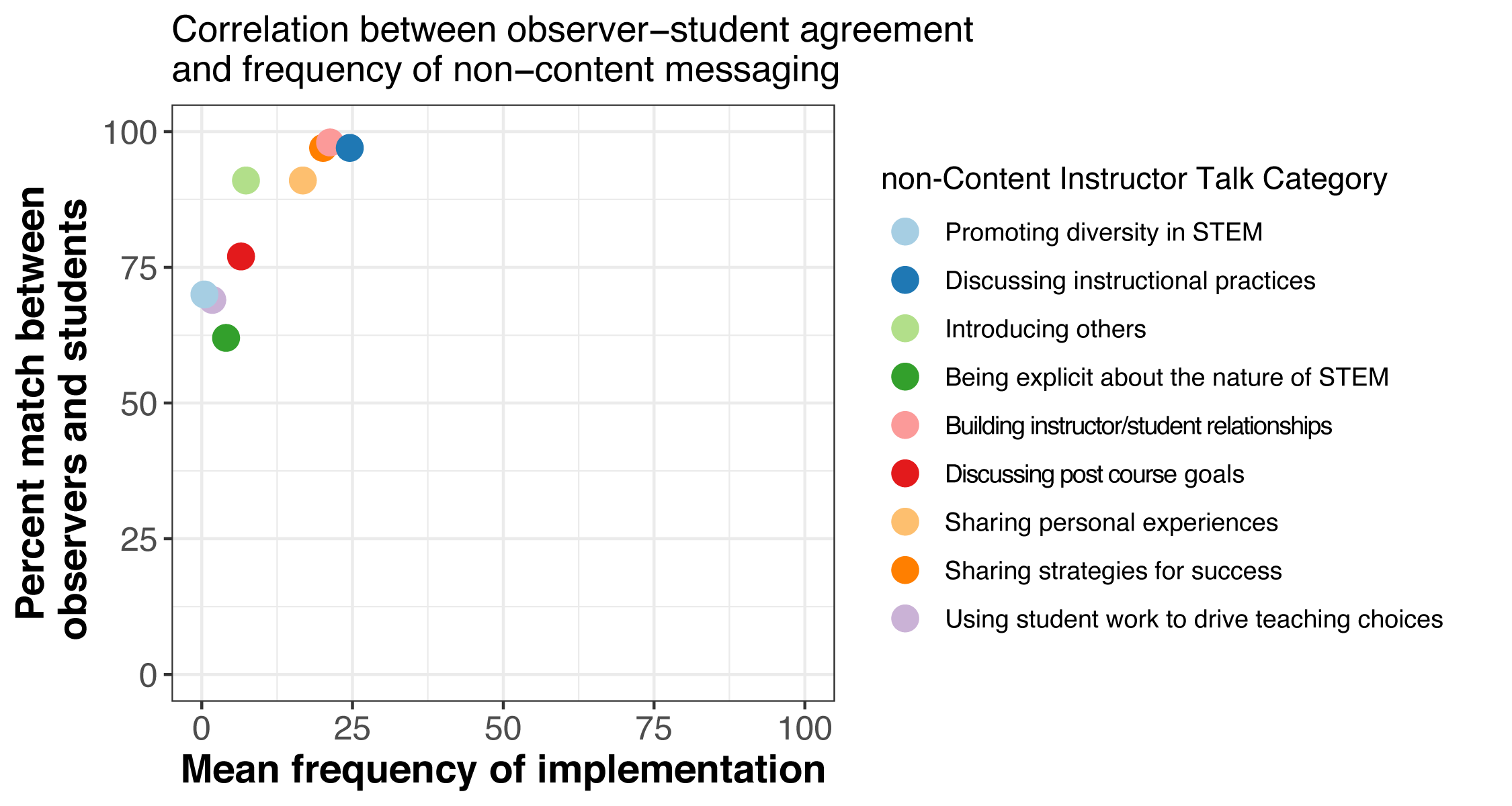


Logistic regression predicting a binary variable (matching or not matching observations) based on students’ demographic variables. * represents *p*  values less than 0.05.

| Summary of logistic regression analysis for variables predicting student survey responses matching observers across all questions | | | |
| --- | --- | --- | --- |
| **Variables** | **B** | **SE** | **Odds Ratio** |
| URM student | 0.184* | 0.07 | 1.20 |

Cross-tabulation of counts based on URM status and whether or not their survey results matched with observations.

|  | URM | non-URM |
| --- | --- | --- |
| Matched with observers | 1989 [85%] | 8073 [83%] |
| Did not match with observers | 342 [15%] | 1656 [17%] |

Cross-tabulation of counts based on URM student status. The counts are organized by whether or not the student survey results matched with observers and are disaggregated by non-content Instructor Talk question category. The two categories highlighted in bold text show the categories where URM students were more likely to match to observers.

|  | URM students | | Non-URM students | |
| --- | --- | --- | --- | --- |
| **Category of non-content Instructor Talk** | **Matched observers** | **Did not match observers** | **Matched observers** | **Did not match observers** |
| Building instructor/student relationships | 251 [97%] | 8 [3%] | 1056 [98%] | 25 [2%] |
| Discussing instructional practices | 252 [97%] | 7 [3%] | 1047 [97%] | 34 [3%] |
| Sharing strategies for success | 247 [95%] | 12 [5%] | 1046 [97%] | 35 [3%] |
| Sharing personal experiences | 240 [93%] | 19 [7%] | 983 [91%] | 98 [9%] |
| Introducing others | 230 [89%] | 29 [11%] | 987 [91%] | 94 [9%] |
| Discussing post-course goals | 205 [79%] | 54 [21%] | 826 [76%] | 255 [24%] |
| Promoting diversity in STEM | 184 [71%] | 75 [29%] | 325 [70%] | 756 [30%] |
| **Using student work to drive teaching choices** | **194 [75%]** | **65 [25%]** | **735 [68%]** | **346 [32%]** |
| **Being explicit about the nature of STEM** | **186 [72%]** | **73 [28%]** | **637 [59%]** | **44 4[41%]** |

**Supplemental Appendix 6**. Heatmap of the percent of one-minute intervals each of the 11 instructors dedicated to the nine non-content Instructor Talk categories. Instructors are ordered by the Lower STEM and Higher STEM content coverage clustering identified in Figure 1A.


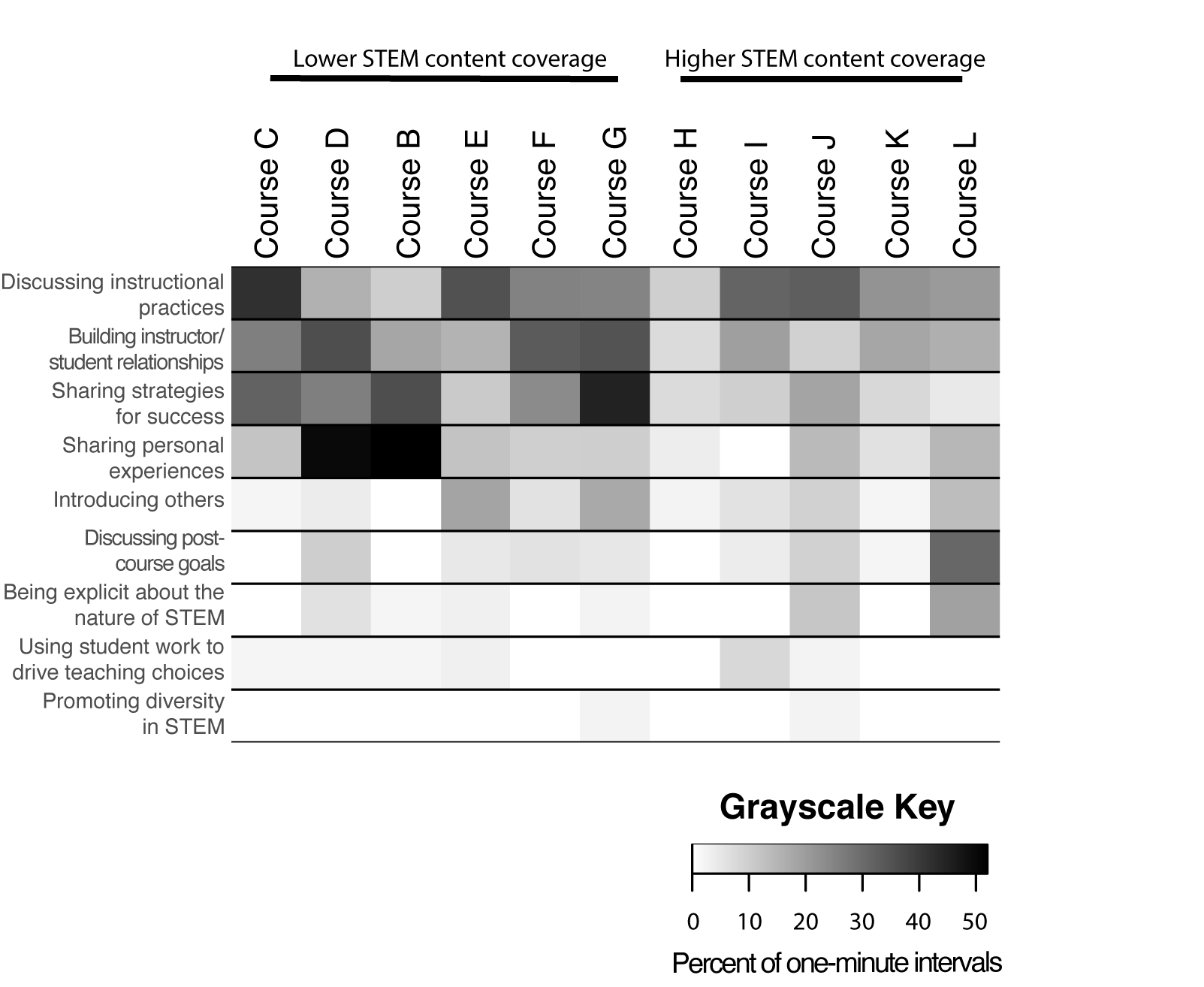

Supplement: Supplementary file 1 — Additional file 1: Appendix S1. Codebook for course structure. Appendix S2. Codebook for non-content Instructor Talk. Appendix S3. Survey questions. Appendix S4. Student demographics. Appendix S5. Statistical tests. Appendix S6. Heatmap of non-content Instructor Talk categories [file 40594_2021_306_MOESM1_ESM.docx]
